# Longitudinal clinical trajectories and vascular–inflammatory–neurophysiological associations in diabetic peripheral neuropathy: an exploratory analysis of a TENS-treated cohort

**DOI:** 10.3389/fendo.2026.1931594

**Published:** 2026-09-03

**Authors:** Qian Gao, Liwan Yang, Bin Wang, Lijuan Gao, Qing Yao, Jiangwei Hu

**Affiliations:** Department of Endocrinology, The Second Affiliated Hospital of Xingtai Medical College, Xingtai, Hebei, China

**Keywords:** Bayesian network, causal inference, diabetic peripheral neuropathy, longitudinal analysis, transcutaneous electrical nerve stimulation, vascular–inflammatory–neurophysiological pathway

## Abstract

**Background:**

Transcutaneous electrical nerve stimulation (TENS) is used as an adjunctive treatment for diabetic peripheral neuropathy (DPN), but long-term clinical trajectories vary among patients. The biological characteristics associated with these different trajectories remain incompletely characterized. This study aimed to identify longitudinal clinical trajectory patterns and examine their associations with vascular, inflammatory, and neurophysiological measures.

**Methods:**

Prospective 24-month data from 685 patients with DPN receiving standardized TENS therapy were analyzed using an integrated framework comprising latent class growth analysis, Elastic Net feature selection, Bayesian network analysis, model-based counterfactual simulation, longitudinal mixed-effects modeling, and time-lagged analyses.

**Results:**

Three longitudinal trajectory classes were identified: Favorable Improvement (29.9%), Moderate Improvement (42.0%), and Persistent High Burden (28.0%). In subsequent binary analyses, the first two classes were combined as the improved group and the Persistent High Burden class as the non-responder group. ABI, IL-6, and SNCV were among the variables most consistently associated with trajectory classification across complementary analyses. Bayesian network analysis identified conditional dependencies among vascular, inflammatory, neurophysiological, and trajectory-related variables. Model-based counterfactual simulations showed progressively lower estimated probabilities of Persistent High Burden under hypothetical modifications of ABI alone, ABI plus IL-6, and ABI plus IL-6 plus SNCV; the estimated probability changed from 42.7% at the observational baseline to 34.2%, 21.8%, and 11.5%, respectively. Longitudinal mixed-effects and time-lagged analyses showed broadly consistent temporal associations among ABI, IL-6, SNCV, and GCS. Sensitivity analyses, including an SNCV-excluded composite outcome, yielded similar trajectory classifications.

**Conclusions:**

Distinct longitudinal clinical trajectories were observed among patients with DPN receiving TENS. ABI, IL-6, and SNCV were consistently associated with trajectory classification and longitudinal GCS measures. The network and counterfactual analyses provide a model-based description of relationships among these domains but do not establish causal effects or clinical benefits from modifying individual factors. These findings may help inform future studies investigating multidomain determinants of long-term DPN trajectories.

## 1 Introduction

Diabetic peripheral neuropathy (DPN) is a common complication of diabetes and a major cause of chronic neuropathic pain, sensory loss, and disability, affecting up to half of people with diabetes (1, 2). Transcutaneous electrical nerve stimulation (TENS) is used as an adjunctive non-pharmacological treatment for DPN symptoms (3, 4). However, clinical responses during long-term follow-up vary among patients receiving TENS (5–7). Some patients experience sustained improvement, whereas others continue to have substantial symptom burden despite the same treatment protocol (5–7). Characterizing the factors associated with these different longitudinal patterns may help refine patient stratification and inform future studies of neuromodulation (8).

Previous studies have mainly examined baseline characteristics, including glycemic and electrophysiological measures, in relation to clinical outcomes (9). Although these measures provide useful information, a single baseline assessment may not capture changes across vascular, inflammatory, and neurophysiological domains during follow-up (10–12). DPN is characterized by abnormalities across multiple biological systems (13), and evaluating these domains separately may not fully describe their relationships over time.

We therefore examined whether different longitudinal clinical trajectories among TENS-treated patients were associated with changes in vascular, inflammatory, and neurophysiological measures (14, 15). Particular attention was given to peripheral perfusion, inflammatory activity, and nerve conduction because these measures represent complementary aspects of DPN. The analyses were designed to characterize their temporal and statistical relationships without assuming a predefined causal sequence.

Using prospective 24-month data from 685 patients with DPN receiving standardized TENS therapy, we combined latent class growth analysis, Elastic Net feature selection, Bayesian network analysis, model-based counterfactual analysis, and longitudinal mixed-effects models. The primary aim was to characterize heterogeneous clinical trajectories and identify baseline and time-varying factors associated with persistent symptom burden. Network and counterfactual analyses were exploratory and were used to further characterize statistical dependencies and generate hypotheses for future controlled studies.

## 2 Methods

### 2.1 Overall study design

This prospective, single-center observational cohort study examined longitudinal clinical trajectories in patients with DPN receiving standardized TENS therapy. The analysis focused on trajectory patterns during follow-up and baseline biological characteristics associated with trajectory classification. The analytical workflow included latent class growth analysis, Elastic Net feature selection, Bayesian network analysis, model-based counterfactual analysis, longitudinal mixed-effects modeling, and sensitivity analyses.

**Figure 1:**
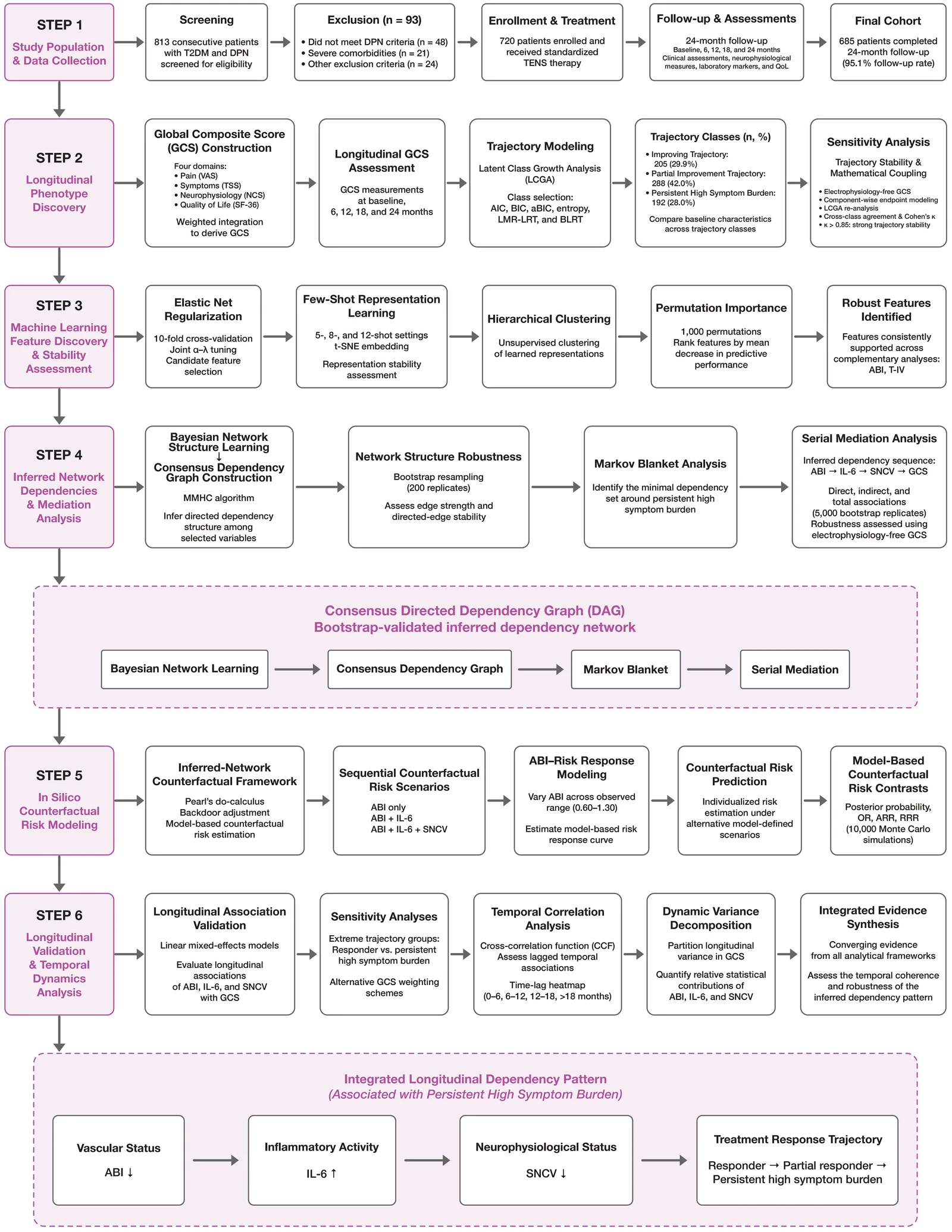
Overall study design and multilevel analytical framework for characterizing longitudinal symptom trajectories in diabetic peripheral neuropathy.

The workflow comprised five steps (**Figure 1**). First, latent class growth analysis (LCGA) was applied to repeated Global Composite Score (GCS) measurements over 24 months to identify distinct longitudinal trajectory classes. Baseline demographic, clinical, biochemical, vascular, inflammatory, and neurophysiological characteristics were then compared across classes.

Second, Elastic Net regression was used to identify baseline variables associated with the Persistent High Burden trajectory. The selected feature space was further examined using t-distributed stochastic neighbor embedding (t-SNE) visualization, hierarchical clustering, and permutation-based feature importance analysis.

Third, Bayesian network structure learning was used to examine conditional dependencies among the selected baseline variables. Markov blanket analysis was performed to identify variables directly connected with trajectory classification, and serial mediation analysis was used to characterize indirect statistical associations involving vascular, inflammatory, and neurophysiological measures.

Fourth, model-based counterfactual analyses were conducted using the learned network structure. Prespecified hypothetical modifications of selected variables were used to estimate changes in the model-implied probability of Persistent High Burden trajectory membership. These analyses were exploratory and were not intended to estimate TENS treatment effects or clinical benefits of modifying the selected variables.

Finally, longitudinal linear mixed-effects models were used to examine associations between trajectory classification, time-varying biological measures, and GCS. Pre-specified sensitivity analyses included an electrophysiology-free composite score to assess potential mathematical coupling between sensory nerve conduction velocity (SNCV) and GCS, alternative GCS weighting schemes, and exclusion of the intermediate trajectory class. Time-lagged cross-correlation analyses were also performed to examine temporal patterns among vascular, inflammatory, neurophysiological, and clinical measures.

### 2.2 Study design and participants

This prospective, single-center observational cohort study was conducted at the Second Affiliated Hospital of Xingtai Medical College. The study examined longitudinal clinical trajectories in patients with DPN receiving TENS therapy according to a standardized treatment protocol. Between January 2022 and March 2024, 813 consecutive patients with T2DM and confirmed DPN were screened for eligibility.

Participants were eligible if they met all of the following criteria: (1) age 18–75 years; (2) T2DM diagnosed according to the 2019 World Health Organization criteria (16); (3) DPN confirmed by a Toronto Clinical Neuropathy Score (TCNS) >5 and electrophysiological evidence of peripheral nerve impairment (17, 18); and (4) baseline HbA1c ≤11.0%.

Patients were excluded for the following reasons: (1) non-diabetic neuropathies, including vitamin B12 deficiency, chronic inflammatory demyelinating polyneuropathy, or alcoholic neuropathy; (2) advanced peripheral artery disease (ABI<0.40) or critical limb ischemia with extensive gangrene requiring immediate revascularization; (3) active lower-extremity ulceration or local skin infection precluding electrode placement; or (4) the presence of a cardiac pacemaker or implantable cardioverter-defibrillator.

Of the 813 screened patients, 720 met the eligibility criteria and initiated TENS therapy. Thirty-five participants were lost to follow-up during the first month, leaving 685 participants in the final analytical cohort.

The study was conducted in accordance with the Declaration of Helsinki and approved by the Institutional Review Board of the Second Affiliated Hospital of Xingtai Medical College (Approval No. 2022KY021). Written informed consent was obtained from all participants before enrollment. The study was reported in accordance with the Strengthening the Reporting of Observational Studies in Epidemiology (STROBE) guidelines.

### 2.3 Treatment protocol and adherence assessment

All participants received standardized TENS using a certified dual-channel device. Trained clinicians applied the electrodes bilaterally at Zusanli (ST36), Sanyinjiao (SP6), and Kunlun (BL60) according to standard anatomical landmarks.

Stimulation was delivered at 20 Hz with a pulse width of 200 μs. Current intensity was individually adjusted to the highest tolerable level without pain, muscle contraction, or marked local discomfort (3). Participants were instructed to complete two 30-minute sessions daily (60 minutes/day) throughout the 24-month follow-up.

Device-recorded session number and duration were used to assess adherence. High adherence was defined *a priori* as completion of ≥ 90% of scheduled sessions. Adherence was summarized descriptively and was not included as a primary analytical covariate.

Concomitant medications, including glucose-lowering agents, insulin, and neuropathic pain medications such as gabapentinoids and tricyclic antidepressants, were recorded at baseline and during follow-up. Medication changes were documented, while diabetes management continued according to routine clinical practice.

### 2.4 Follow-up and outcome measures

Participants were followed for 24 months after TENS initiation, with assessments at baseline and 1, 3, 6, 12, 18, and 24 months. Measurements at baseline, 6, 12, 18, and 24 months were used for trajectory analyses.

The primary outcome was the longitudinal trajectory of the GCS. Secondary outcomes included pain intensity, neuropathy symptom severity, peripheral nerve electrophysiology, health-related quality of life, and safety outcomes.

Pain intensity was assessed using a 10-cm Visual Analog Scale (VAS), and neuropathy symptom severity using the Total Symptom Score (TSS). Peripheral nerve function was assessed by SNCV and motor nerve conduction velocity (MNCV) using standardized neurophysiological procedures. Health-related quality of life was assessed using the Physical Component Summary (PCS) of the 36-Item Short Form Health Survey (SF-36). GCS calculation and weighting are described in Section 2.5.

Clinical and neurophysiological assessments were performed by trained investigators using standardized procedures. Assessors were unaware of participants’ TENS adherence records and trajectory assignments. The same clinical team performed assessments at successive visits whenever possible.

Safety outcomes were recorded at each follow-up visit. Device-related adverse events, including local skin irritation, contact dermatitis, and electrical discomfort, were summarized descriptively.

### 2.5 Construction of the global composite score

A weighted GCS was used to summarize longitudinal changes in pain, neuropathy symptoms, peripheral nerve conduction, and health-related quality of life. The GCS at each follow-up visit (*t*) was calculated as:


$$\text{GCS}={\text{w}}_{1}\text{ ΔVA}{\text{S}}_{t}\%+{\text{w}}_{2}\text{ ΔTS}{\text{S}}_{t}\%+{\text{w}}_{3}\text{ ΔNC}{\text{S}}_{t}\%+{\text{w}}_{4}\text{ ΔQo}{\text{L}}_{t}\%$$

where Δ% represents the percentage change from baseline at visit t. ΔVAS_t% and ΔTSS_t% represent the percentage changes in pain intensity and neuropathy symptom severity, respectively. The electrophysiological component (ΔNCS*_t_*%) was calculated as the mean percentage change in sensory and motor nerve conduction velocities:.


$$\Delta \text{NCSt}\%=\frac{\Delta \text{SNCVt}\%+\Delta \text{MNCVt}\%}{2}$$

ΔQoL*_t_*% represented the percentage change in the Physical Component Summary of the SF-36.

The weights were prespecified according to clinical domain importance. Pain and neuropathy symptoms each contributed 25% of the total score (w1 = 0.25 and w2 = 0.25), while neurophysiological measures and quality of life contributed 30% and 20%, respectively (w3 = 0.30 and w4 = 0.20). Thus, the four domain weights summed to 1.0.

The direction of each percentage change was harmonized so that higher values indicated greater improvement. The weighted GCS was calculated first, and the resulting GCS values were subsequently standardized using *Z* scores before LCGA.

For descriptive analyses only, GCS values were classified into three levels: marked improvement (GCS ≥30.0%), moderate improvement (10.0% ≤ GCS<30.0%), and minimal or no improvement (GCS<10.0%). These categories were used only for descriptive comparisons and were not used in LCGA or subsequent inferential analyses.

### 2.6 Baseline assessments and data management

Baseline demographic, clinical, biochemical, neurophysiological, and treatment-related data were collected before TENS initiation using standardized case report forms. Demographic and clinical variables included age, sex, body mass index (BMI), diabetes duration, and DPN symptom duration. Clinical phenotypes were classified according to symptom presentation and electrophysiological findings.

Laboratory assessments included glycated hemoglobin (HbA1c), fasting lipid profiles, estimated glomerular filtration rate (eGFR), high-sensitivity C-reactive protein (hs-CRP), interleukin-6 (IL-6), and the neutrophil-to-lymphocyte ratio (NLR). Laboratory measurements were performed in the hospital’s central clinical laboratory using routine procedures. Vascular status was assessed by diabetic retinopathy (DR) screening, ankle-brachial index (ABI) measurement, and Doppler ultrasonography of lower-extremity arterial plaques. Neuropathy symptoms, electrophysiological measures, and health-related quality of life were assessed using the methods described in Section 2.4.

All baseline assessments were performed by trained investigators. Data were checked for completeness and consistency during collection and before analysis.

Missing data were assessed for all analytical variables. Missing baseline covariates were imputed using multiple imputation by chained equations (MICE), with relevant demographic, biochemical, and longitudinal variables included in the imputation models (19, 21). Twenty imputed datasets were generated and combined using Rubin’s rules (22). Longitudinal analyses were conducted using linear mixed-effects models, with available repeated measurements retained for analysis (20). Continuous covariates were standardized as Z scores before Elastic Net feature selection and longitudinal modeling.

### 2.7 Statistical analysis

#### 2.7.1 Longitudinal trajectory modelling and baseline stratification

LCGA was performed to identify longitudinal clinical trajectories among TENS-treated patients using repeated GCS measurements at baseline and 6, 12, 18, and 24 months (23). Models with one to four classes and linear or quadratic growth functions were evaluated (24). Model selection considered AIC, BIC, classification entropy (>0.80), and the Lo–Mendell–Rubin adjusted likelihood ratio test (LMR-LRT). Classes containing fewer than 5% of the cohort (*N* = 685) were not considered (25). Participants were assigned to the class with the highest posterior membership probability, and mean posterior probabilities were used to assess classification quality (23, 24).

Baseline characteristics were then compared across trajectory classes. Continuous variables are presented as mean ± standard deviation or median [interquartile range (IQR)] and were compared using one-way analysis of variance (ANOVA) or the Kruskal–Wallis test, as appropriate. Categorical variables are presented as number (percentage) and were compared using the chi-square test or Fisher’s exact test.

#### 2.7.2 Machine learning feature discovery and stability assessment

For the binary analysis, Classes 1 and 2 were combined as the improved group (*n* = 493), and Class 3 was defined as the non-responder group (*n* = 192). Elastic Net regression was applied to standardized baseline demographic, clinical, laboratory, vascular, and neurophysiological variables (26, 27). The mixing parameter was fixed at *α* = 0.50. The penalty parameter *λ* was selected by 10-fold cross-validation using both *λ_min_* and *λ_1s_*_e_ criteria (27).

t-SNE was used to visualize the selected feature space (28). Repeated 5-shot, 8-shot, and 12-shot sampling analyses were performed to evaluate the stability of classification under different sample sizes. Hierarchical clustering with Ward.D2 linkage was used to characterize the resulting sample structure. Permutation feature importance was calculated from 1,000 random permutations, and the resulting feature rankings were compared with the standardized Elastic Net coefficients (29).

#### 2.7.3 Bayesian network architecture and mediation analysis

Bayesian network analysis was used to characterize conditional dependencies among the selected baseline variables without imposing a predefined causal structure (30, 31). The network was learned using the Max–Min Hill-Climbing (MMHC) algorithm (32). Network stability was evaluated by non-parametric bootstrap resampling with 200 replicates. Edges with both edge strength and directional consistency greater than 0.85 were retained in the consensus directed acyclic graph (DAG) (31).

Markov blanket analysis was used to identify variables directly connected with trajectory classification in the learned network (30). Serial mediation analysis was performed to quantify the direct and indirect statistical associations involving baseline ABI, IL-6, SNCV, and trajectory classification. Direct, indirect, and total effects were estimated using 5,000 bootstrap replications, with 95% bias-corrected confidence intervals (33). These analyses were interpreted as statistical associations and were not used to establish a causal pathway.

#### 2.7.4 Model-based counterfactual analysis using *do*-calculus

Exploratory counterfactual analyses were conducted using the consensus DAG and Pearl’s do-calculus framework (34). The analysis examined changes in the model-estimated probability of the Persistent High Burden trajectory under hypothetical changes in selected biological variables. Three scenarios were specified: (1) ABI set to the prespecified normal range; (2) ABI and IL-6 simultaneously set to their prespecified target states; and (3) ABI, IL-6, and SNCV simultaneously set to their prespecified target states.

For each scenario, posterior outcome probabilities were estimated from 10,000 Monte Carlo draws (30). Model-based odds ratios (ORs), absolute risk differences (ARDs), and relative risk differences (RRDs) were calculated against the observational baseline state. The modeled probability of Persistent High Burden was also evaluated across the observed ABI range (0.60–1.30). Representative high-, intermediate-, and low-risk baseline profiles were used to illustrate differences in model-estimated probabilities under the specified scenarios (35).

These analyses describe counterfactual quantities implied by the learned DAG and its underlying assumptions. They were not treated as estimates of clinical treatment effects or as evidence of a confirmed causal relationship among ABI, IL-6, and SNCV.

#### 2.7.5 Longitudinal validation, sensitivity analysis, and dynamic time-lag decomposition

Linear mixed-effects (LME) models were fitted to repeated GCS measurements over the 24-month follow-up. Fixed effects included follow-up time, LCGA trajectory class, their interaction, and time-varying ABI, IL-6, and SNCV, together with baseline diabetes duration and clinical phenotype. Subject-specific random intercepts accounted for within-participant correlation, and models were estimated using restricted maximum likelihood (REML).

Two prespecified sensitivity analyses were performed. First, the LME models were re-fitted after excluding the Moderate Improvement class to assess the stability of the longitudinal associations in the two extreme trajectory groups. Second, alternative GCS weighting schemes were examined using the objective-heavy GCS-A and subjective-heavy GCS-B specifications. The direction and statistical significance of the main longitudinal associations were compared across weighting schemes; AIC and BIC were not used to select a preferred weighting scheme because the weighting schemes represented different outcome definitions.

Time-lagged cross-correlation functions were used to examine temporal relationships among ABI, IL-6, SNCV, and GCS across four follow-up intervals (0 – 6, 6 – 12, 12 – 18, and >18 months). Dynamic variance decomposition was then used to partition the observed longitudinal change in GCS into components associated with changes in ABI, IL-6, and SNCV.

#### 2.7.6 Sensitivity analyses for trajectory stability and mathematical coupling verification

To assess the stability of the trajectory classification and address potential mathematical coupling between baseline SNCV and longitudinal GCS, two pre-specified sensitivity analyses were performed. First, an SNCV-excluded composite score (ΔGCS_exSNCV_) was constructed by removing the SNCV component from the primary GCS and renormalizing the remaining component weights.


$$\Delta \text{GCSexSNCV,t}=\frac{0.25\;.\Delta \text{VASt}\%+0.25\;.\;\Delta \text{TSSt}\%+0.20\;.\;\Delta \text{QoLt}\%}{0.70}$$

LCGA was then repeated using ΔGCS_exSNCV,_*_t_* at the same follow-up time points and with the same model-selection criteria as the primary analysis. Agreement between the primary GCS-based and SNCV-excluded trajectory classifications was assessed using cross-tabulation and Cohen’s *κ*. A *κ* > 0.85 was prespecified to indicate high classification stability.

Second, component-wise longitudinal models were fitted separately for ΔVAS%, ΔTSS%, ΔSNCV%, and ΔQoL%, and baseline associations with the SNCV-excluded trajectory phenotypes were re-evaluated. Baseline SNCV was retained as a candidate predictor to assess whether its association persisted after removal from the composite outcome. Consistent trajectory classification (*κ* > 0.85) and preserved baseline associations were considered supportive of robustness against mathematical coupling.

#### 2.7.7 Computational environment and software

All analyses were performed in R version 4.3.2 (R Foundation for Statistical Computing, Vienna, Austria). LCGA was conducted using “lcmm”; Elastic Net regression and related analyses using “glmnet” and “caret”; Bayesian network and bootstrap analyses using “bnlearn” and “boot”; linear mixed-effects models using “lme4”; and t-SNE visualization using “Rtsne”. All statistical tests were two-sided, with *P* < 0.05 considered statistically significant.

## 3 Results

### 3.1 Identification of longitudinal trajectory patterns and multidimensional symptom heterogeneity

**Figure 2:**
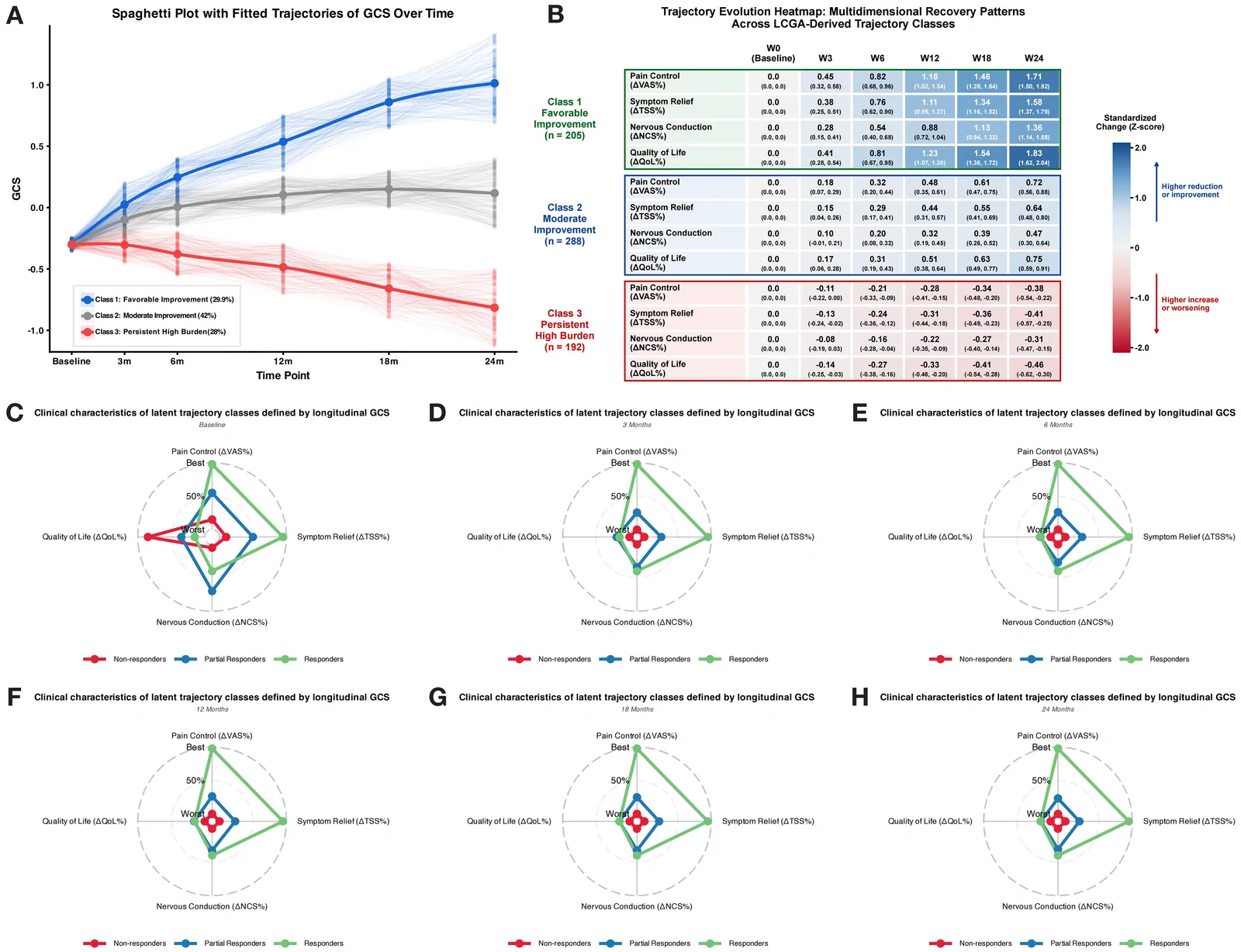
Latent Trajectory Classes Based on Serial Global Composite Scores LCGA classified patients with DPN receiving standardized TENS therapy into three trajectory classes over 24 months. **(A)** Longitudinal GCS trajectories. Individual observations and fitted curves for Class 1 (Favorable Improvement; *n* = 205, 29.9%), Class 2 (Moderate Improvement; *n* = 288, 42.0%), and Class 3 (Persistent High Burden; *n* = 192, 28.0%). Thick lines indicate class-specific mean trajectories with 95% CIs. **(B)** Changes in GCS domains. Standardized changes in pain control (ΔVAS%), symptom relief (ΔTSS%), neurophysiological function (ΔNCS%), and quality of life (ΔQoL%) at each follow-up assessment. Higher values indicate greater improvement. **(C–H)** Multidomain profiles by trajectory class. Radar plots at baseline **(C)**, 3 months **(D)**, 6 months **(E)**, 12 months **(F)**, 18 months **(G)**, and 24 months **(H)** showing the four GCS domains across the three trajectory classes. DPN, diabetic peripheral neuropathy; GCS, Global Composite Score; LCGA, latent class growth analysis; NCS, nerve conduction component; QoL, quality of life; TENS, transcutaneous electrical nerve stimulation; TSS, total symptom score; VAS, visual analog scale.

LCGA based on serial GCS measurements over the 24-month follow-up identified three distinct longitudinal trajectory classes among the participants (**Figure 2A**). Class 1 (Favorable Improvement; *n* = 205, 29.9%) displayed a sustained increase in GCS throughout follow-up. Class 2 (Moderate Improvement; *n* = 288, 42.0%) showed greater improvement during the first 12 months followed by a relative plateau. In contrast, Class 3 (Persistent High Burden; *n* = 192, 28.0%) demonstrated a progressive decline in GCS over time.

Standardized longitudinal profiles of pain control (ΔVAS%), symptom severity (ΔTSS%), sensory nerve conduction (ΔSNCV%), and quality of life (ΔQoL%) further characterized multidimensional differences across trajectory classes (**Figures 2B–H**). Class 1 showed broadly concordant improvement across domains. Class 2 showed greater gains in patient-reported measures (ΔVAS% and ΔTSS%) than in ΔSNCV%, whereas Class 3 showed deterioration across multiple domains, particularly in pain and neurophysiological measures.

In the prespecified sensitivity analysis, LCGA was re-executed using the electrophysiology-free composite score (ΔGCS_exSNCV_). Classification agreement between the primary and sensitivity models was 92.6%, with high concordance by Cohen’s *κ* (*κ* = 0.915, *P* < 0.001; **Supplementary Figure 1**). The high consistency of trajectory assignment after removal of the SNCV component supports the stability of the identified longitudinal patterns and reduces concern that trajectory classification was substantially influenced by mathematical coupling between SNCV and GCS.

### 3.2 Baseline phenotypic differences across trajectories

Baseline characteristics differed across the three LCGA-derived trajectory classes (**Table 1**). Demographic variables, including age (*P* = 0.290), sex (*P* = 0.891), and body mass index (*P* = 0.211), were similar across groups. In contrast, several measures of metabolic status differed between the Favorable Improvement and Persistent High Burden trajectories. Compared with Class 1, patients in Class 3 had higher glycated hemoglobin levels (8.3 ± 1.4% vs. 7.4 ± 0.9%, P< 0.001), longer diabetes duration (14.5 vs. 9.0 years, *P* < 0.001), and more frequent insulin use (48.4% vs. 33.2%, *P* = 0.006).

**Table 1:** Baseline characteristics of patients according to longitudinal GCS trajectory classes (*N* = 685).

Baseline characteristics	Total cohort (N = 685)	Class 1: favorable improvement (n = 205)	Class 2: moderate improvement (n = 288)	Class 3: persistent high burden (n = 192)	Test statistic	*P_value_*
Demographic & General Clinical Features						
Age (years), mean ± SD	62.4 ± 8.7	61.8 ± 8.2	62.3 ± 8.9	63.2 ± 9.1	1.24*δ*	0.290
Male, n (%)	390 (56.9)	114 (55.6)	166 (57.6)	110 (57.3)	0.23*^b^*	0.891
BMI (kg/m²), mean ± SD	25.8 ± 3.4	25.5 ± 3.2	25.9 ± 3.5	26.1 ± 3.6	1.56*δ*	0.211
Diabetes duration (years), median [IQR]	11.5 [7.0, 16.5]	9.0 [5.5, 14.0]	12.0 [7.5, 17.0]	14.5 [9.5, 19.5]	28.43*^c^*	<0.001
DPN symptom duration (months), median [IQR]	24.0 [12.0, 48.0]	18.0 [10.0, 36.0]	24.0 [12.0, 48.0]	36.0 [18.0, 60.0]	34.12*^c^*	<0.001
DPN Severity Stratification & Clinical Phenotypes						
Painful DPN (VAS ≥3 and DN4 ≥4), n (%)	445 (65.0)	162 (79.0)	190 (66.0)	93 (48.4)	41.25*^b^*	<0.001
Painless DPN (Numbness/coldness without pain), n (%)	240 (35.0)	43 (21.0)	98 (34.0)	99 (51.6)		
Sensory-dominant phenotype (Pure sensory NCS damage), n (%)	260 (38.0)	104 (50.7)	112 (38.9)	44 (22.9)	32.84*^b^*	<0.001
Mixed sensory-motor phenotype (Combined NCS damage), n (%)	425 (62.0)	101 (49.3)	176 (61.1)	148 (77.1)		
Glycemic & Basic Metabolic Indices						
HbA1c (%), mean ± SD	7.8 ± 1.2	7.4 ± 0.9	7.8 ± 1.1	8.3 ± 1.4	32.15*δ*	<0.001
Triglycerides (mmol/L), median [IQR]	1.85 [1.40, 2.45]	1.72 [1.31, 2.20]	1.88 [1.42, 2.48]	1.98 [1.50, 2.65]	8.54*^c^*	0.014
eGFR (mL/min/1.73 m²), mean ± SD	84.2 ± 15.6	87.1 ± 13.8	84.5 ± 15.2	80.6 ± 17.3	10.42*δ*	<0.001
Biological & Inflammatory Axis						
hs-CRP (mg/L), median [IQR]	2.10 [1.15, 3.80]	1.55 [0.90, 2.70]	2.15 [1.20, 3.75]	2.95 [1.60, 5.20]	42.67*^c^*	<0.001
IL-6 (pg/mL), median [IQR]	3.45 [2.10, 5.80]	2.30 [1.45, 3.80]	3.50 [2.25, 5.65]	4.95 [3.10, 8.40]	78.14*^c^*	<0.001
NLR	2.35 [1.75, 3.10]	1.95 [1.50, 2.55]	2.40 [1.80, 3.15]	2.80 [2.05, 3.90]	39.51*^c^*	<0.001
Microvascular Axis Status						
DR, n (%)	215 (31.4)	42 (20.5)	92 (31.9)	81 (42.2)	22.56*^b^*	<0.001
ABI, mean ± SD	1.04 ± 0.12	1.09 ± 0.09	1.04 ± 0.11	0.98 ± 0.14	45.12*δ*	<0.001
Lower-extremity arterial plaque formation, n (%)	310 (45.3)	72 (35.1)	132 (45.8)	106 (55.2)	17.43*^b^*	<0.001
Baseline Outcome Sources (Neural Axis/TENS Benchmarks)						
Baseline VAS pain score, mean ± SD	5.8 ± 1.6	5.6 ± 1.5	5.8 ± 1.6	6.1 ± 1.7	3.89*δ*	0.021
TSS, mean ± SD	6.4 ± 1.9	6.1 ± 1.7	6.4 ± 1.9	6.9 ± 2.1	6.12*δ*	0.002
SNCV (m/s), mean ± SD	38.5 ± 4.6	40.2 ± 4.1	38.4 ± 4.4	36.1 ± 4.8	38.74*δ*	<0.001
Sural nerve action potential amplitude (μV), mean ± SD	4.2 ± 1.8	5.1 ± 1.9	4.1 ± 1.7	3.1 ± 1.4	48.21*δ*	<0.001
Baseline Quality of Life (SF-36 Physical Component Summary)	44.2 ± 8.5	46.5 ± 7.8	44.0 ± 8.4	41.3 ± 9.1	18.45*δ*	<0.001
Concomitant Medication & TENS Parameters						
Insulin therapy, n (%)	285 (41.6)	68 (33.2)	124 (43.1)	93 (48.4)	10.15*^b^*	0.006
Aldose reductase inhibitor (Epalrestat), n (%)	340 (49.6)	108 (52.7)	145 (50.3)	87 (45.3)	2.14*^b^*	0.343
Baseline gabapentinoids/tricyclics use, n (%)	152 (22.2)	36 (17.6)	65 (22.6)	51 (26.6)	4.92*^b^*	0.085
Standardized TENS compliance (Session rate ≥ 90%), n (%)	634 (92.6)	192 (93.7)	266 (92.4)	176 (91.7)	0.62*^b^*	0.733

Data are presented as mean ± SD, median [IQR], or number (percentage).

^a^ One-way ANOVA (F statistic) for normally distributed continuous variables.

^b^ Pearson *χ²* test (*χ²* statistic) for categorical variables.

^c^ Kruskal-Wallis H test (H statistic) for non-normally distributed continuous variables. ABI, ankle-brachial index; BMI, body mass index; DN4, Douleur Neuropathique 4; DPN, diabetic peripheral neuropathy; DR, diabetic retinopathy; eGFR, estimated glomerular filtration rate; HbA_1c_, glycated hemoglobin; hs-CRP, high-sensitivity C-reactive protein; IL-6, interleukin-6; NCS, nerve conduction studies; NLR, neutrophil-to-lymphocyte ratio; SF-36, 36-Item Short Form Health Survey; SNCV, sensory nerve conduction velocity; TENS, transcutaneous electrical nerve stimulation; TSS, total symptom score; VAS, visual analog scale.

Inflammatory and vascular measures also differed between the two groups. Median IL-6 levels (4.95 vs. 2.30 pg/mL, *P<* 0.001) and neutrophil-to-lymphocyte ratios (2.80 vs. 1.95, *P* < 0.001) were higher in Class 3. Class 3 also had lower ABI values (0.98 ± 0.14 vs. 1.09 ± 0.09], *P* < 0.001), a higher prevalence of lower-extremity arterial plaque (55.2% vs. 35.1%, *P* < 0.001), and a higher prevalence of diabetic retinopathy (42.2% vs. 20.5%, *P* < 0.001).

Neurophysiological measures showed greater impairment in Class 3. Painful symptoms were less common in this group than in Class 1 (48.4% vs. 79.0%, *P* < 0.001), whereas the mixed sensory-motor subtype was more frequent (77.1% vs. 49.3%, *P* < 0.001). Baseline sensory nerve conduction velocity was lower in Class 3 (36.1 ± 4.8 vs. 40.2 ± 4.1 m/s, *P* < 0.001), as was sural nerve action potential amplitude (3.1 ± 1.4 vs. 5.1 ± 1.9 μV, *P* < 0.001). Physical quality-of-life scores were also lower in Class 3 (41.3 ± 9.1, P< 0.001).

Background medication use and high TENS adherence (≥ 90%) were similarly distributed across the trajectory classes (*P* = 0.733). Thus, adherence did not differ significantly between the trajectory groups.

### 3.3 Machine learning–based feature selection and stability assessment

For the binary analysis, Classes 1 and 2 were combined into the improved group (*n* = 493), and Class 3 was defined as the non-responder group (*n* = 192).

**Figure 3:**
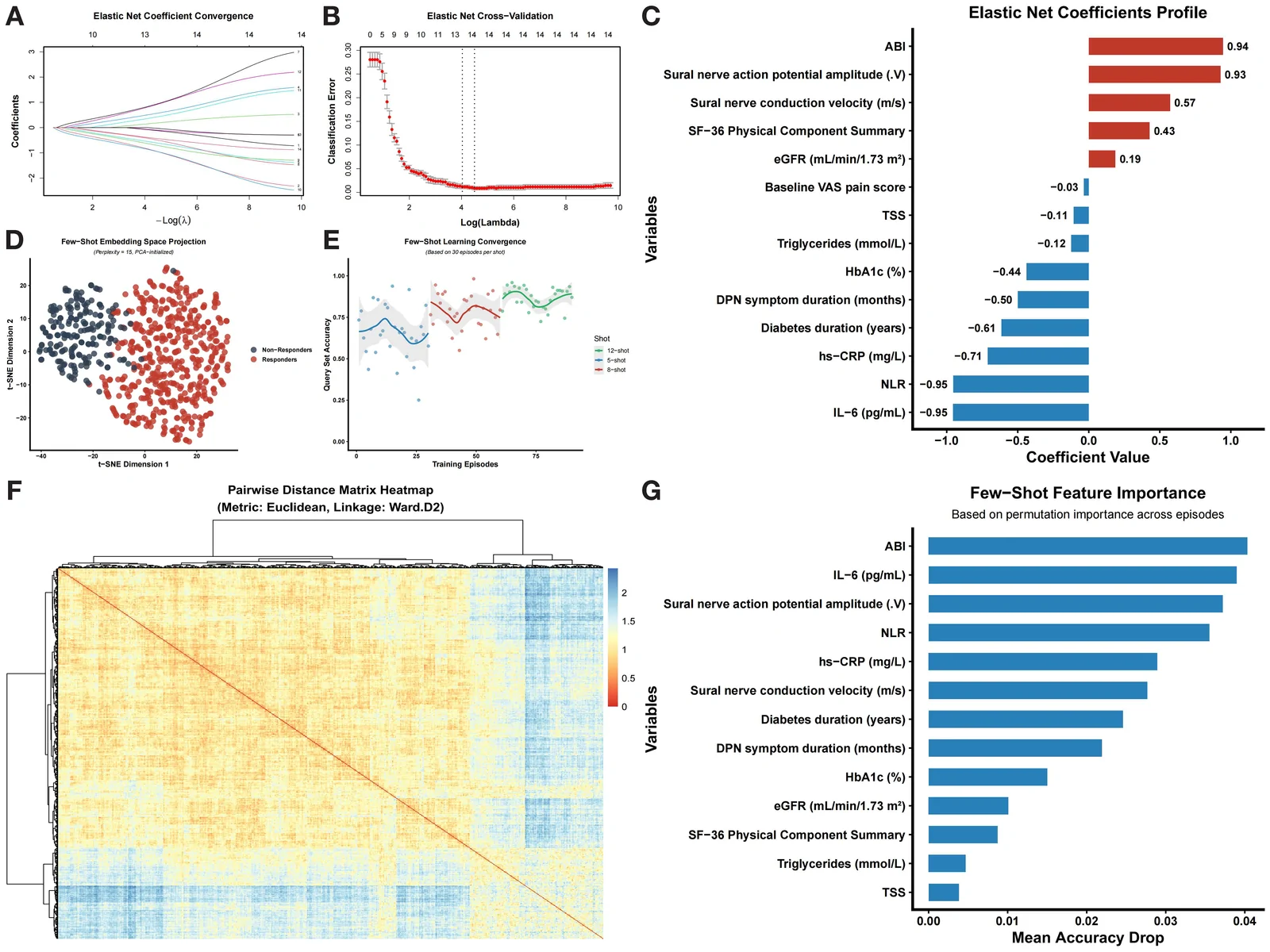
Regularized Feature Selection and Stability Assessment for Trajectory Classification Elastic Net regularization, sample-constrained stability analyses, hierarchical clustering, and permutation-based feature importance were used to evaluate baseline features associated with longitudinal trajectory classification. For binary classification, Classes 1 and 2 (Favorable and Moderate Improvement) were combined as the improved group, while Class 3 (Persistent High Burden) was defined as the non-responder group. **(A, B)** Elastic Net feature selection. **(A)** Coefficient profiles across increasing values of the regularization parameter (−log[*λ*]). **(B)** Ten-fold cross-validation profiles showing classification error across log(*λ*), with vertical lines indicating *λ_min_* and *λ_1se_*. **(C)** Elastic Net coefficient profiles. Standardized baseline variables ranked by the magnitude of their Elastic Net coefficients. The sign indicates the direction of association according to the outcome coding. **(D)** t-SNE representation of the selected feature space. Two-dimensional projection showing the distribution of participants based on selected baseline features, with partial separation and residual overlap between the improved and non-responder groups. **(E)** Sample-constrained stability analysis. Classification consistency across repeated 5-shot, 8-shot, and 12-shot sampling analyses, assessing the stability of feature selection and group discrimination under limited-sample conditions. **(F)** Distance matrix and hierarchical clustering. Pairwise distance structure and Ward.D2 clustering based on selected baseline features, illustrating participant similarity and subgroup organization. **(G)** Permutation-based feature importance. Reduction in classification performance after 1,000 random permutations of individual variables. Larger reductions indicate greater importance for the observed classification performance. ABI, ankle-brachial index; eGFR, estimated glomerular filtration rate; HbA1c, glycated hemoglobin; hs-CRP, high-sensitivity C-reactive protein; IL-6, interleukin-6; NLR, neutrophil-to-lymphocyte ratio; SF-36, 36-Item Short Form Health Survey; t-SNE, t-distributed stochastic neighbor embedding; λ, regularization tuning parameter.

Elastic Net was applied to the baseline variables, with ten-fold cross-validation used to select *λ_min_* and *λ_1se_* (**Figures 3A, B**). Higher ABI, SNAP amplitude, SNCV, and baseline SF-36 Physical Component Summary scores were associated with the improved group. In contrast, IL-6, NLR, hs-CRP, and longer diabetes duration showed greater association with the non-responder group (**Figure 3C**).

The t-SNE plot showed partial separation between the two groups, with overlap among individual observations (**Figure 3D**). Repeated 5-shot, 8-shot, and 12-shot analyses yielded different levels of classification consistency, with the highest consistency in the 12-shot analysis (**Figure 3E**). Hierarchical clustering based on the selected variables showed similar subgroup patterns, although substantial variation remained within each trajectory group (**Figure 3F**).

Permutation analysis ranked ABI as the most important feature, followed by IL-6, SNAP amplitude, and NLR (**Figure 3G**). These features were also among those retained or ranked highly by the Elastic Net analysis.

The selected baseline variables were then used in the Bayesian network analysis to examine their conditional dependencies and associations with trajectory classification.

### 3.4 Probabilistic network topology and serial pathway mediation

**Figure 4:**
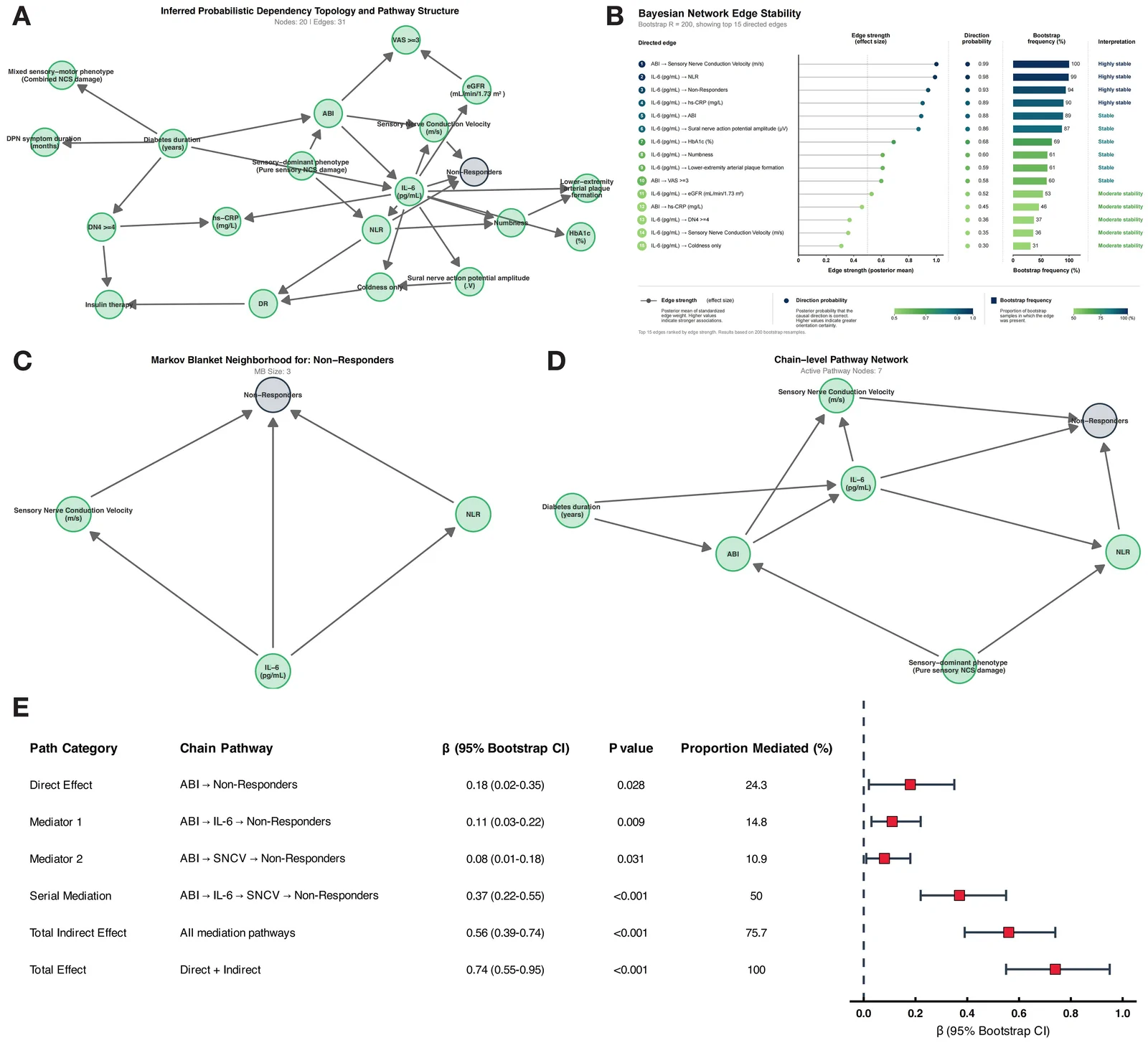
Bayesian Network Structure and Serial Mediation Analysis of the Persistent High Burden Trajectory Bayesian network and serial mediation analyses were used to examine conditional dependencies and statistical associations among vascular, inflammatory, neurophysiological, and clinical variables related to the Persistent High Burden trajectory. **(A)** Bayesian network structure. Consensus DAG learned using the MMHC algorithm, showing 31 directed edges among 23 baseline variables. The network captures conditional dependencies among vascular, inflammatory, neurophysiological, and clinical features. **(B)** Network stability. Non-parametric bootstrap resampling (200 replicates) was used to assess edge strength and directional consistency. The 15 most stable directed edges are shown. **(C)** Markov blanket of the Persistent High Burden trajectory. Local network structure highlighting IL-6, NLR, and SNCV as variables directly connected with the Persistent High Burden trajectory node. **(D)** Vascular–inflammatory–neurophysiological subnetwork. Focused network showing the conditional dependencies among ABI, IL-6, SNCV, and trajectory classification. The network structure represents statistical dependencies and does not establish causality. **(E)** Serial mediation analysis. Bootstrap analysis (5,000 iterations) decomposing the association between baseline ABI and Persistent High Burden trajectory membership into direct and indirect components. Estimates are presented with 95% CIs. The combined indirect component accounted for 75.7% of the total estimated association, including the sequential ABI–IL-6–SNCV pathway. These estimates represent statistical indirect associations rather than evidence of a causal pathway ABI, ankle-brachial index; CI, confidence interval; DAG, directed acyclic graph; IL-6, interleukin-6; MMHC, Max–Min Hill-Climbing; NLR, neutrophil-to-lymphocyte ratio; SNCV, sensory nerve conduction velocity.

Structure learning identified a consensus DAG comprising 23 nodes and 31 directed edges, describing conditional dependencies among the selected baseline variables (**Figure 4A**). The network included interconnected inflammatory, vascular, neurophysiological, and clinical variables. Bootstrap resampling (R = 200) showed consistent recovery of the network structure, with the 15 most stable edges showing relatively high edge strength and directional consistency (**Figure 4B**).

The Markov blanket of the Persistent High Burden trajectory included IL-6, NLR, and SNCV (**Figure 4C**). A focused subnetwork further showed conditional dependencies among ABI, inflammatory markers, neurophysiological measures, and trajectory classification (**Figure 4D**).

Serial mediation analysis was then used to characterize the statistical associations among baseline ABI, IL-6, SNCV, and Persistent High Burden trajectory membership (**Figure 4E**). Lower ABI was associated with Persistent High Burden trajectory membership (total association *β* = 0.74, 95% CI: 0.55 – 0.95, *P<* 0.001). The estimated direct component accounted for 24.3% of the total association (*β* = 0.18, 95% CI: 0.02 – 0.35, *P* = 0.028), whereas the combined indirect component accounted for 75.7% (*β* = 0.56, 95% CI: 0.39 – 0.74, *P* < 0.001). The largest individual indirect component was the sequential association involving ABI, IL-6, and SNCV (*β* = 0.37, 95% CI: 0.22 – 0.55, P< 0.001), accounting for 50.0% of the total association.

Overall, the network and mediation analyses showed interconnected statistical relationships among vascular, inflammatory, and neurophysiological measures and Persistent High Burden trajectory membership. These analyses were observational and do not establish a causal pathway. The identified variables were subsequently examined in the model-based counterfactual analyses.

### 3.5 Exploratory *in silico* counterfactual risk simulations

**Figure 5:**
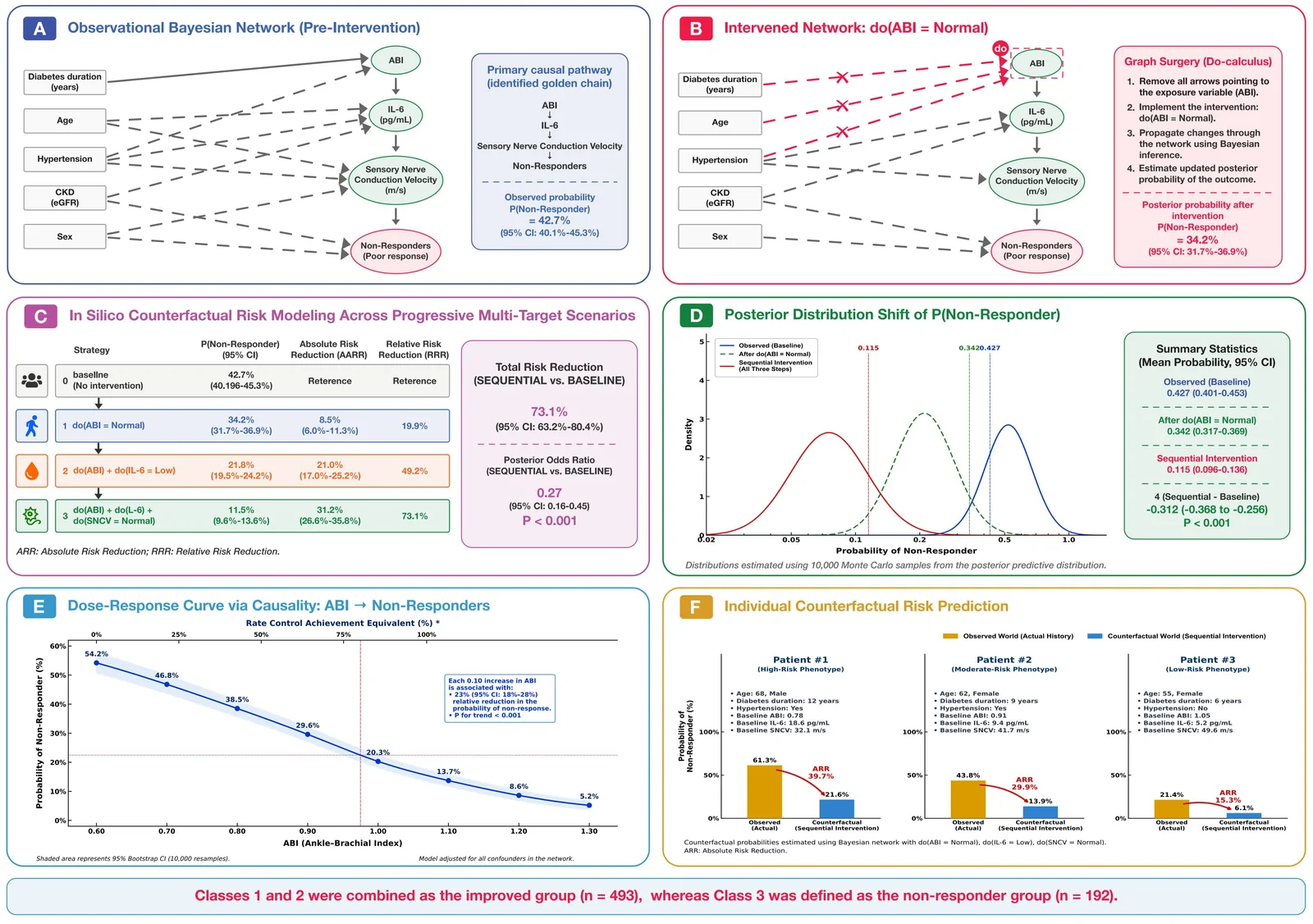
Model-Based Counterfactual Simulations Under Sequential Hypothetical Interventions. Counterfactual simulations based on the consensus Bayesian network examined changes in the model-estimated probability of the Persistent High Burden trajectory under hypothetical modifications of ABI, IL-6, and SNCV. These estimates represent model-implied differences and should not be interpreted as clinical treatment effects. **(A)** Observational Bayesian network showing the dependencies among ABI, IL-6, SNCV, trajectory classification, and other baseline variables. The estimated probability of Persistent High Burden trajectory membership was 42.7% (95% CI, 40.1% – 45.3%). **(B)** Network after do(ABI = normal), with incoming edges to ABI removed according to the intervention rule. The estimated probability decreased to 34.2% (95% CI, 31.7%–36.9%). **(C)** Model-estimated probabilities under sequential hypothetical interventions on ABI, ABI+IL-6, and ABI+IL-6+SNCV were 34.2%, 21.8%, and 11.5%, respectively. The three-variable scenario corresponded to an absolute risk difference of −31.2 percentage points and a model-based relative risk difference of 73.1% (95% CI, 63.2% – 80.4%; OR, 0.27; 95% CI, 0.16 – 0.45). **(D)** Posterior probability distributions from 10,000 Monte Carlo simulations under the observational, ABI-only, and three-variable scenarios. The mean probability changed from 0.427 to 0.115 under the three-variable scenario (difference, −0.312; 95% CI, −0.368 to −0.256). **(E)** Model-estimated probability of Persistent High Burden trajectory membership across the observed ABI range (0.60 – 1.30). A 0.10-unit increase in ABI corresponded to a 23% relative difference in modeled trajectory risk (95% CI, 18%–28%; *P_trend<_* 0.001). This represents a model-based association rather than a causal dose–response effect. **(F)** Individualized counterfactual simulations for three representative risk profiles. Under the three-variable scenario, estimated probabilities decreased from 61.3% to 21.6%, 43.8% to 13.9%, and 21.4% to 6.1% in high-, intermediate-, and low-risk profiles, respectively. ABI, ankle-brachial index; CI, confidence interval; DAG, directed acyclic graph; *do*, intervention operator in Pearl’s do-calculus; IL-6, interleukin-6; OR, odds ratio; SNCV, sensory nerve conduction velocity.

Pearl’s *do*-calculus was applied to the consensus DAG to estimate model-implied changes in the probability of Persistent High Burden trajectory membership under prespecified hypothetical modifications of ABI, IL-6, and SNCV (**Figures 5A–F**). For each scenario, the corresponding node was graphically intervened on by removing its incoming edges.

Under the observational state, the estimated probability of Persistent High Burden trajectory membership was 42.7% (95% CI: 40.1 – 45.3%). Setting ABI to the prespecified normal range reduced the model-estimated probability to 34.2% (95% CI: 31.7 – 36.9%), an absolute difference of −8.5 percentage points (95% CI: −11.3 to −6.0). Joint modification of ABI and IL-6 yielded an estimated probability of 21.8% (95% CI: 19.5 – 24.2%), while simultaneous modification of ABI, IL-6, and SNCV yielded 11.5% (95% CI: 9.6 – 13.6%), corresponding to an absolute difference of −31.2 percentage points (95% CI: −35.8 to −26.6) relative to the observational state. The corresponding model-based relative difference was 73.1% (95% CI: 63.2 – 80.4%), with an OR of 0.27 (95% CI: 0.16 – 0.45; *P* < 0.001).

Monte Carlo simulation produced a similar distributional shift in the three-variable scenario, with the mean estimated probability changing from 0.427 to 0.115 (absolute difference, −0.312; 95% CI: −0.368 to −0.256; **Figure 5D**). Across the ABI range of 0.60–1.30, the model-estimated probability also showed an inverse gradient (*P_trend_* < 0.001; **Figure 5E**), with an estimated 23% relative difference for each 0.10-unit increase in ABI (95% CI: 18 – 28%). Patient-level simulations showed variation in the magnitude of model-estimated probability differences across representative baseline profiles, ranging from 15.3 to 39.7 percentage points under the three-variable scenario (**Figure 5F**).

These estimates represent model-implied counterfactual differences under the assumptions encoded in the learned DAG, rather than observed treatment effects or expected clinical benefits from modifying ABI, IL-6, or SNCV.

### 3.6 Longitudinal consistency of network-related associations

**Figure 6:**
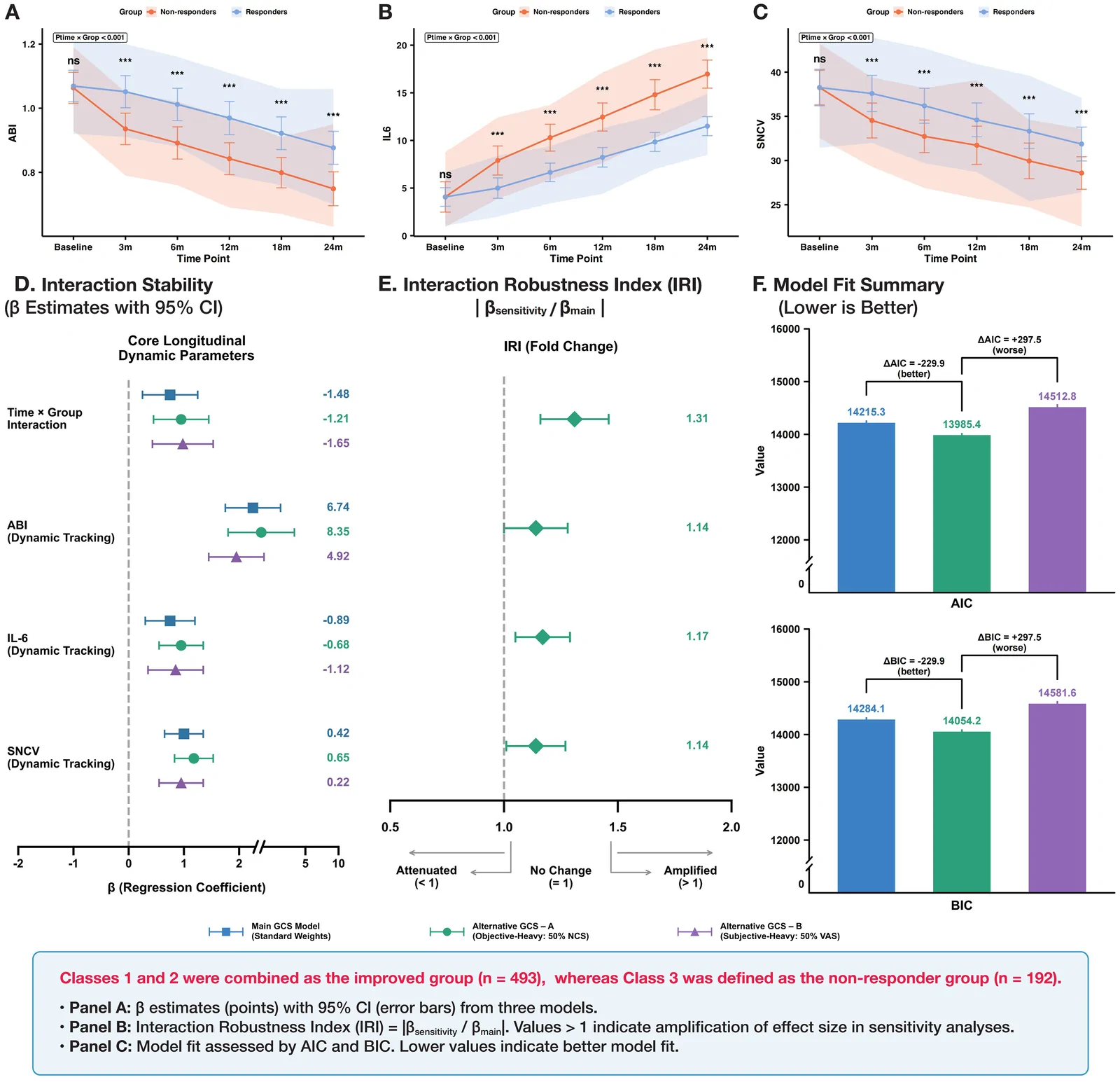
Longitudinal Profiles of Key Biological Variables and Sensitivity Analyses of the Longitudinal Models. Longitudinal changes in ABI, IL-6, and SNCV and sensitivity analyses assessing the consistency of the time-by-trajectory interaction across alternative model specifications. **(A–C)** Longitudinal trajectories of **(A)** ABI, **(B)** IL-6, and **(C)** SNCV over 24 months in the Favorable Improvement (Class 1) and Persistent High Burden (Class 3) groups. Shaded areas represent 95% CIs. Time-by-group interactions were significant for all three variables (*P_time×group<_*0.001). **(D)** Comparison of time-by-trajectory interaction coefficients across the primary balanced GCS model and alternative objective-heavy (GCS-A) and subjective-heavy (GCS-B) weighting schemes. Estimates are presented as *β* with 95% CIs. **(E)** IRI comparing the interaction coefficient in the sensitivity model with that of the primary model. Values closer to 1 indicate greater similarity in effect magnitude. **(F)** Model fit comparisons based on AIC and BIC across the three GCS specifications. Lower values indicate better relative model fit. ABI, ankle-brachial index; AIC, Akaike Information Criterion; BIC, Bayesian Information Criterion; CI, confidence interval; GCS, Global Composite Score; IL-6, interleukin-6; IRI, Interaction Ratio Index; SNCV, sensory nerve conduction velocity.

Longitudinal changes in ABI, IL-6, and SNCV were examined over 24 months (**Figures 6A–C**). ABI declined more markedly in the Persistent High Burden class than in the Favorable Improvement class, with a significant time-by-class interaction (*P_time×group_* < 0.001). IL-6 remained higher and showed greater between-class separation over follow-up in the Persistent High Burden class (*P_time×group_<* 0.001). SNCV also declined more rapidly in this class (*P_time×group_<* 0.001).

Linear mixed-effects models including all participants (*N* = 685) were fitted using restricted maximum likelihood (**Table 2**). After adjustment, GCS was lower in the Persistent High Burden class than in the Favorable Improvement class (*β* = −4.82, 95% CI: −6.95 to −2.69, *P* < 0.001). GCS increased over follow-up overall (*β* = 2.35, 95% CI: 1.88 – 2.82, *P* < 0.001), but the increase was smaller in the Persistent High Burden class (time × class: *β* = −1.48, 95% CI: −1.92 to −1.04, *P* < 0.001).

**Table 2:** Linear mixed-effects model predicting GCS across 24 months (*N* = 685).

Parameter/fixed & random components	Estimate (β)	95% CI	SE	*t_Value_*	*P_Value_*
Fixed Effects					
Intercept (Favorable trajectory reference at Month 3)	12.45	10.12, 14.78	1.19	10.46	< 0.001
Trajectory group (Persistent High Burden vs. Favorable)	-4.82	-6.95, -2.69	1.09	-4.42	< 0.001
Follow-up time (per 3-month increment)	2.35	1.88, 2.82	0.24	9.79	< 0.001
Time × Persistent High Burde group	-1.48	-1.92, -1.04	0.22	-6.73	< 0.001
Time-Varying Covariates (Repeated Measures)					
Longitudinal ABI	6.74	4.12, 9.36	1.34	5.03	< 0.001
Longitudinal IL-6 (pg/mL)	-0.89	-1.24, -0.54	0.18	-4.94	< 0.001
Longitudinal SNCV (m/s)	0.42	0.21, 0.63	0.11	3.82	< 0.001
Baseline Adjustment Covariates					
Age (per year)	-0.05	-0.14, 0.04	0.05	-1	0.317
Diabetes duration (per year)	-0.12	-0.23, -0.01	0.06	-2	0.046
Sensory-dominant phenotype (vs. mixed sensory-motor)	2.15	0.88, 3.42	0.65	3.31	0.001
Baseline gabapentinoid/tricyclic use	-0.34	-1.12, 0.44	0.4	-0.85	0.396
Random Effects (Variance Components)	Variance	95% CI			
Random intercept variance	14.25	10.11, 18.39	—	—	—
Random slope variance (Time)	1.18	0.73, 1.63	—	—	—
Residual variance	8.64	7.76, 9.52	—	—	—
Model Fit Statistics					
AIC	14215.3	—	—	—	—
BIC	14284.1	—	—	—	—

Estimates were obtained from a linear mixed-effects model with subject-specific random intercepts and random slopes for follow-up time, fitted using REML. Follow-up time was modeled continuously and centered at 3 months, with coefficients expressed per 3-month increment. The Improved group (Classes 1 and 2) served as the reference group, and the Persistent High Burden group (Class 3) was modeled as the comparison group. Time-varying ABI, IL-6, and SNCV were entered as repeated measures, while baseline age, diabetes duration, clinical phenotype, and baseline gabapentinoid/tricyclic use were included as adjustment covariates. Regression coefficients (*β*) are unstandardized estimates. Because SNCV contributes to the electrophysiological component of the GCS, its association with GCS may be subject to mathematical coupling and was therefore interpreted as a component-level longitudinal association rather than independent prognostic evidence; its robustness was assessed in the prespecified electrophysiology-free sensitivity analysis. ABI, ankle-brachial index; AIC, Akaike Information Criterion; BIC, Bayesian Information Criterion; CI, confidence interval; GCS, Global Composite Score; IL-6, interleukin-6; SE, standard error; SNCV, sensory nerve conduction velocity.

In models including time-varying covariates, higher ABI was associated with higher GCS (*β* = 6.74, 95% CI: 4.12 – 9.36, *P* < 0.001), whereas higher IL-6 was associated with lower GCS (*β* = −0.89, 95% CI: −1.24 to −0.54, *P* < 0.001). SNCV was positively associated with GCS (*β* = 0.42, 95% CI: 0.21–0.63, *P* < 0.001). Longer diabetes duration (*β* = −0.12, *P* = 0.046) and sensory-dominant clinical phenotype (*β* = 2.15, *P* = 0.001) also remained associated with GCS.

Overall, the longitudinal models showed persistent associations of ABI, IL-6, and SNCV with GCS during follow-up, consistent with their relationships with trajectory classification and the network analysis. These results describe longitudinal statistical associations and do not establish a causal relationship among the three biological measures.

### 3.7 Robustness assessment across cohort subsets and outcome formulations

Prespecified sensitivity analyses examined the stability of the primary findings under alternative trajectory definitions, GCS weighting schemes, and an electrophysiology-free outcome.

First, an electrophysiology-free composite score (ΔGCS_exSNCV_) was constructed to assess potential mathematical coupling between SNCV and GCS. LCGA was repeated using the same assessment time points and model-selection criteria as the primary analysis. Trajectory assignments showed 92.6% agreement with the primary classification (Cohen’s *κ* = 0.915, *P* < 0.001; **Supplementary Figure 1**). Component-level analyses of ΔVAS%, ΔTSS%, ΔSNCV%, and ΔQoL% showed similar longitudinal patterns.

Second, the Moderate Improvement class was excluded, leaving 397 participants in the Favorable Improvement and Persistent High Burden classes (**Table 3**). The time × trajectory group interaction changed from *β* = −1.48 to *β* = −1.94, with a 31.1% increase in absolute coefficient magnitude (IRI = 1.31, *P* < 0.001; **Figures 6D–E**). Longitudinal ABI (*β* = 7.12), IL-6 (*β* = −1.04), and SNCV (*β* = 0.48) remained significant and directionally consistent with the primary model (all *P* ≤ 0.002).

**Table 3:** Sensitivity analysis 1: linear mixed-effects models comparing extreme trajectory subgroups (excluding moderate-improvement class, *N* = 397).

Parameter/covariate	Primary model (full cohort, N = 685)	Sensitivity model (extreme classes, N = 397)	Δβ (absolute shift)	Δβ% (relative shift)	*P _Value_* (sensitivity)
Core Fixed Effects					
Intercept (Favorable trajectory reference)	12.45	14.12	+1.67	+13.4%	< 0.001
Trajectory group (Persistent High Burden vs. Favorable)	-4.82	-6.15	-1.33	+27.6%	< 0.001
Follow-up time (per 3-month increment)	2.35	2.68	+0.33	+14.0%	< 0.001
Time × Trajectory group interaction	-1.48	-1.94	-0.46	+31.1%	< 0.001
Time-Varying Covariates (Repeated Measures)					
Longitudinal ABI	6.74	7.12	+0.38	+5.6%	< 0.001
Longitudinal IL-6 (pg/mL)	-0.89	-1.04	-0.15	+16.9%	< 0.001
Longitudinal SNCV (m/s)	0.42	0.48	+0.06	+14.3%	0.002

The sensitivity analysis excluded the Moderate Improvement trajectory (Class 2; n = 288), leaving 397 participants in the two extreme trajectory classes: Favorable Improvement (Class 1; n = 205) and Persistent High Burden (Class 3; n = 192). The primary and sensitivity models used REML with random intercepts and random slopes for follow-up time and included the same baseline covariates. Δ*β* denotes the absolute coefficient difference, calculated as *β_sensitivity_* − *β_primary_*. Δ*β%* denotes the relative magnitude of coefficient change, calculated as |*β_sensitivity_* − *β_primary_*|/|*β_primary_*| × 100\%. The exploratory IRI, defined as |*β_sensitivity_*/*β_primar_*|, was 1.31 for the time × trajectory group interaction. AIC was 10,188.42 for the sensitivity model and 14,215.30 for the primary model; because the models were fitted to different samples, these AIC values were not interpreted as a direct measure of improved model fit. ABI, ankle-brachial index; AIC, Akaike Information Criterion; IL-6, interleukin-6; IRI, interaction ratio index; REML, restricted maximum likelihood; SNCV, sensory nerve conduction velocity.

Third, alternative GCS weighting schemes were evaluated (**Table 4**). With objective-heavy weighting (GCS-A), the time × trajectory group interaction was *β* = −1.21 (*P* < 0.001), with longitudinal ABI, IL-6, and SNCV coefficients of 8.35 (*P* < 0.001), −0.68 (*P* = 0.001), and 0.65 (*P* < 0.001), respectively. With subjective-heavy weighting (GCS-B), the corresponding estimates were −1.65 (*P* < 0.001), 4.92 (*P* = 0.002), −1.12 (*P* < 0.001), and 0.22 (*P* = 0.041)(**Figure 6F**). Thus, coefficient magnitudes varied across weighting schemes, whereas the directions of the main longitudinal associations were unchanged.

**Table 4:** Sensitivity analysis 2: linear mixed-effects models across alternative composite outcome weighting schemes (N = 685).

Parameter/covariate	Primary model (balanced weighting)	Alternative model A (objective-heavy)	Alternative model B (subjective-heavy)
Core Longitudinal Parameters			
Time × Trajectory group interaction, β (P value)	−1.48 (<0.001)	−1.21 (<0.001)	−1.65 (<0.001)
Longitudinal ABI, β (P value)	6.74 (<0.001)	8.35 (<0.001)	4.92 (0.002)
Longitudinal IL-6, β (P value)	−0.89 (<0.001)	−0.68 (0.001)	−1.12 (<0.001)
Longitudinal SNCV, β (P value)	0.42 (<0.001)	0.65 (<0.001)	0.22 (0.041)

Sensitivity analyses used alternative GCS weighting schemes with the same linear mixed-effects model as the primary analysis. The primary GCS assigned 25% each to VAS pain and TSS, 30% to the neurophysiological component (mean percentage change in SNCV and MNCV), and 20% to the SF-36 Physical Component Summary. GCS-A assigned 50% to the neurophysiological component and 16.7% each to VAS, TSS, and QoL; GCS-B assigned 50% to VAS and 16.7% each to the neurophysiological component, TSS, and QoL.

All models used REML with random intercepts and slopes for follow-up time and the same baseline covariates and time-varying parameters as the primary model. The analysis assessed whether the main longitudinal associations were preserved under different GCS definitions. AIC and BIC were not used to compare weighting schemes because they represented different outcome formulations. The time × trajectory group, longitudinal ABI, and longitudinal IL-6 associations remained directionally consistent and statistically significant across models. The longitudinal SNCV association remained positive, with differences in effect size and statistical significance across weighting schemes. ABI, ankle-brachial index; GCS, Global Composite Score; IL-6, interleukin-6; MNCV, motor nerve conduction velocity; QoL, quality of life; REML, restricted maximum likelihood; SNCV, sensory nerve conduction velocity; TSS, total symptom score; VAS, visual analog scale.

Overall, the trajectory classification and principal longitudinal associations were similar across the alternative outcome and subgroup specifications. The high agreement between the primary and SNCV-excluded classifications indicates that the trajectory structure was preserved after removing SNCV from the composite score.

### 3.8 Time-lagged cross-correlations and dynamic variance decomposition

**Figure 7:**
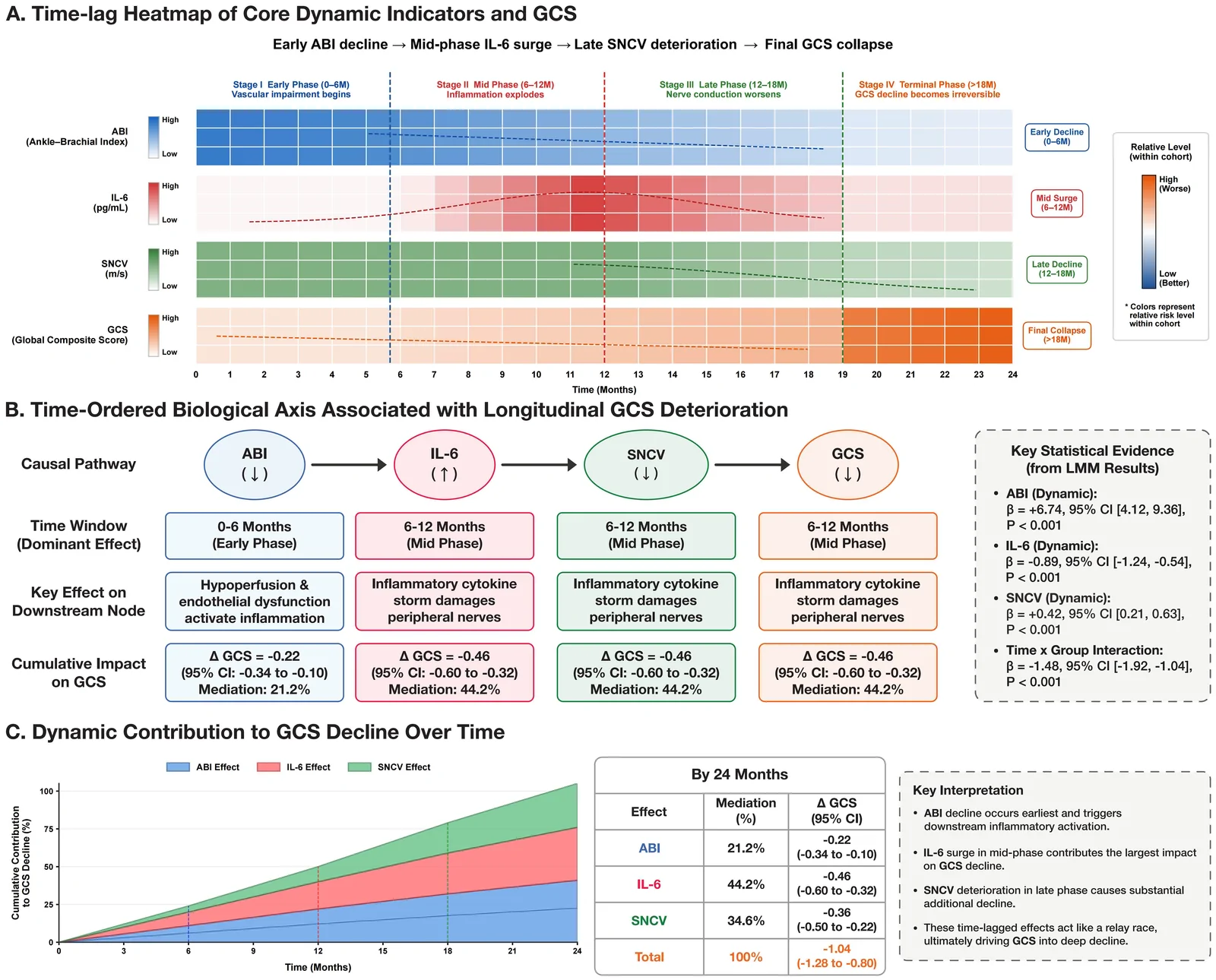
Time-Lagged Associations and Sequential Pathways of Longitudinal GCS Trajectories Time-lagged analyses were used to examine the temporal ordering of changes in microvascular, inflammatory, and neurophysiological measures in relation to longitudinal GCS trajectories. **(A)** Time-Lagged Cross-Correlation Heatmap. Cross-correlation coefficients across four follow-up intervals (0 – 6, 6 – 12, 12 – 18, and > 18 months), showing an early shift in ABI, followed by changes in IL-6, SNCV, and later GCS divergence. **(B)** Time-Ordered Pathway. Sequential associations among ABI, IL-6, SNCV, and GCS based on time-lagged longitudinal modeling. Standardized path coefficients indicate the direction and magnitude of associations between consecutive nodes. **(C)** Variance Decomposition of Longitudinal GCS Change. The cumulative GCS change over 24 months was −1.04 (95% CI, −1.28 to −0.80). Changes in ABI, IL-6, and SNCV accounted for 21.2%, 44.2%, and 34.6% of the estimated GCS change, respectively. ABI, ankle-brachial index; CI, confidence interval; GCS, Global Composite Score; IL-6, interleukin-6; SNCV, sensory nerve conduction velocity.

Time-lagged cross-correlation analyses examined temporal associations among ABI, IL-6, SNCV, and GCS during the 24-month follow-up (**Figures 7A–C**). The strongest cross-correlation signals occurred at different follow-up intervals: ABI changes were most prominent during 0–6 months, IL-6 during 6–12 months, SNCV during 12–18 months, and GCS toward the later follow-up period (**Figure 7A**). The resulting lag structure showed that these measures were not synchronized over time and broadly corresponded to the ABI – IL-6 – SNCV – GCS ordering observed in the network and longitudinal analyses (**Figure 7B**).

Dynamic variance decomposition was used to partition the observed longitudinal change in GCS according to the modeled contributions of ABI, IL-6, and SNCV. The mean total change in GCS was −1.04 (95% CI, −1.28 to −0.80). The decomposed components were 21.2% for ABI (ΔGCS = −0.22; 95% CI, −0.34 to −0.10), 44.2% for IL-6 (ΔGCS = −0.46; 95% CI, −0.60 to −0.32), and 34.6% for SNCV (ΔGCS = −0.36; 95% CI, −0.50 to −0.22; **Figure 7C**).

Overall, the analyses showed distinct timing of changes across the vascular, inflammatory, neurophysiological, and clinical measures. The lag structure was compatible with the statistical relationships identified in the other analyses, but does not establish a causal sequence among these variables.

### 3.9 Integrated summary of the multilevel predictive framework

LCGA identified three distinct 24-month trajectories among patients with DPN receiving TENS. The classes differed in clinical, vascular, inflammatory, and neurophysiological measures. For subsequent binary analyses, Classes 1 and 2 were combined as the improved group, and Class 3 was classified as the non-responder group.

ABI, IL-6, and SNCV were consistently associated with trajectory classification in Elastic Net, permutation, Bayesian network, and longitudinal mixed-effects analyses. These associations were largely preserved across alternative trajectory definitions, GCS weighting schemes, and the SNCV-excluded composite outcome (ΔGC_SexSNCV_). Time-lagged analyses showed that changes in these measures occurred at different stages of follow-up, while counterfactual analyses yielded different model-estimated probabilities under specified hypothetical interventions.

Overall, ABI, IL-6, and SNCV were consistently associated with persistent symptom burden. These results describe statistical and temporal relationships rather than a confirmed causal pathway or treatment effect. Whether these measures can improve risk stratification or whether their modification is accompanied by changes in clinical trajectories requires confirmation in independent prospective studies.

## 4 Discussion

### 4.1 Principal findings

This study identified substantial heterogeneity in 24-month clinical trajectories among patients with DPN receiving TENS, despite similar treatment adherence. Three trajectory classes were identified, and the Favorable Improvement and Moderate Improvement classes were combined for subsequent binary analyses, with the Persistent High Burden class defined as the non-responder group.

Compared with the Favorable Improvement class, patients in the Persistent High Burden class had greater baseline metabolic, vascular, inflammatory, and neurophysiological abnormalities, together with less favorable longitudinal patterns of ABI, IL-6, SNCV, and GCS. These findings suggest that differences in treatment trajectories were accompanied by distinct baseline biological profiles and longitudinal changes across multiple clinical domains, rather than apparent differences in treatment adherence.

Across the feature-selection analyses, ABI, IL-6, and SNCV were consistently associated with trajectory classification. Bayesian network analysis further identified conditional dependencies among these variables and trajectory membership, while serial mediation analysis indicated that the association between ABI and Persistent High Burden membership included indirect statistical components involving IL-6 and SNCV. These analyses describe patterns of statistical dependence and indirect association; they do not establish a temporal or causal pathway among vascular, inflammatory, and neurophysiological factors.

Model-based counterfactual analyses showed that hypothetical changes in ABI, IL-6, and SNCV were associated with different estimated probabilities of Persistent High Burden membership. These estimates depend on the assumptions and structure of the learned DAG and should not be interpreted as treatment effects or causal effect estimates. Consistent patterns were also observed across mixed-effects models, alternative GCS weighting schemes, the SNCV-excluded composite score, and time-lagged analyses.

Overall, the findings indicate that persistent symptom burden after TENS was associated with a combination of vascular, inflammatory, and neurophysiological features. These associations may help identify patients with less favorable longitudinal trajectories and provide hypotheses for future prospective studies examining whether modification of these factors is accompanied by changes in treatment response.

### 4.2 Biological context of the vascular–inflammatory–neurophysiological associations

The longitudinal trajectories observed in this study were associated with abnormalities across vascular, inflammatory, and neurophysiological domains rather than with a single clinical measure. ABI, IL-6, and SNCV were consistently associated with trajectory classification or longitudinal GCS, suggesting that these domains may capture complementary aspects of clinical heterogeneity in DPN.

ABI was repeatedly selected in the feature-selection analyses and remained associated with trajectory classification and longitudinal GCS. Adequate endoneurial blood flow is required for axonal metabolism and Schwann cell function, and impaired perfusion may contribute to endoneurial hypoxia, metabolic stress, and impaired nerve repair (1, 36, 37). Metabolic disturbances, including insulin resistance and altered PI3K/Akt signaling, may further contribute to vascular and peripheral tissue dysfunction (38). Thus, lower ABI may represent a broader vascular and metabolic burden associated with less favorable clinical trajectories.

IL-6 was also prominent in the network analysis and was associated with longitudinal GCS. Chronic metabolic and ischemic stress can promote endothelial activation and inflammatory signaling, while elevated IL-6 has been linked to oxidative stress, axonal injury, and altered neuronal function (10, 14, 39). These observations provide a biological context for the association between higher IL-6 levels and less favorable trajectories in this cohort. However, the present analyses cannot determine whether IL-6 represents a mediator of vascular dysfunction, a parallel marker of systemic disease, or both.

SNCV remained associated with trajectory classification and longitudinal GCS. In the lagged analyses, changes in SNCV tended to occur after the observed changes in ABI and IL-6. Although this temporal ordering is compatible with a sequence in which neurophysiological changes follow vascular and inflammatory alterations, it does not establish causality (9, 40). Importantly, the association of baseline SNCV with trajectory classification remained after excluding SNCV from the composite outcome, reducing the likelihood that this finding was primarily attributable to mathematical coupling.

Overall, the findings support an association between vascular, inflammatory, and neurophysiological abnormalities and long-term clinical trajectories in DPN. The observed relationships are biologically plausible and temporally structured, but the data do not establish a vascular–inflammatory–neurophysiological causal cascade. These findings may therefore be useful for identifying patients with less favorable trajectories and for generating hypotheses for prospective studies evaluating whether changes in these biological domains are accompanied by differences in long-term treatment response (1, 14, 15, 35).

### 4.3 Clinical implications and risk stratification

The findings suggest that clinical assessment of DPN may benefit from considering vascular, inflammatory, and neurophysiological measures alongside conventional symptom assessment. In this cohort, differences across these domains were associated with distinct longitudinal trajectories, supporting the potential value of multidomain profiling for identifying patients with a higher likelihood of persistent symptom burden.

Among the baseline measures, ABI may be useful for risk stratification because it is non-invasive and readily available in clinical practice. Lower baseline ABI was consistently associated with the Persistent High Burden trajectory across several analyses. Its assessment may therefore help identify patients with greater vascular burden who could benefit from closer clinical evaluation and management of relevant vascular and metabolic risk factors (1).

The graph-based counterfactual analyses showed different model-estimated probabilities of Persistent High Burden membership under hypothetical modifications of ABI, IL-6, and SNCV. These estimates are conditional on the assumptions and structure of the learned DAG and should not be interpreted as treatment effects. Their main value is hypothesis generation, particularly regarding whether changes across multiple biological domains are associated with different clinical trajectories.

Patient-specific simulations further illustrated how multivariable models could be used to estimate heterogeneity in predicted trajectory risk. However, such applications would require external validation, calibration, and evaluation of clinical utility before they could be considered for clinical use (35).

Overall, these findings support multidomain risk stratification rather than a specific precision-treatment strategy. Prospective studies are needed to determine whether the identified vascular, inflammatory, and neurophysiological factors are modifiable and whether changes in these measures are associated with subsequent improvement in long-term clinical trajectories.

### 4.4 Strengths and limitations

The study has several notable features. The use of latent class modeling together with regularized regression, Bayesian network analysis, and longitudinal mixed-effects models allowed trajectory heterogeneity to be examined using different analytical approaches. Repeated assessments over 24 months also made it possible to evaluate changes in ABI, IL-6, and SNCV alongside changes in GCS, rather than relying only on baseline measurements. The network and counterfactual analyses provided additional information on the relationships among these variables, although their interpretation remains dependent on the underlying model assumptions (35).

There are also important limitations. First, this was a prospective, single-center observational study, and all participants received TENS without an untreated, sham, or alternative-treatment group. The identified trajectories therefore cannot be used to estimate the causal effect of TENS or to conclude that patients differed in their response to TENS itself. Changes during follow-up could also reflect the natural progression of DPN, concomitant treatment, changes in disease management, regression to the mean, or factors not captured in the analysis. The results are consequently best interpreted as different longitudinal patterns observed among patients receiving TENS.

Second, SNCV contributed to the GCS and was also examined as a predictor of trajectory membership, raising the possibility of mathematical coupling. When SNCV was removed from the composite score, the resulting trajectory classification remained highly concordant with the primary classification. This finding makes it less likely that the overall trajectory structure was determined solely by the shared SNCV component, but it does not completely eliminate the possibility of coupling. Results involving SNCV should therefore be interpreted in conjunction with the sensitivity analyses.

Third, the 24-month follow-up may not capture longer-term changes in DPN. In addition, the single-center setting and the characteristics of the study population may limit the applicability of the findings to other clinical settings. Replication in independent, preferably multicenter cohorts is needed before the trajectory classification or associated models can be generalized.

Finally, the Bayesian network, mediation, and do-calculus analyses were performed using observational data and rely on assumptions specified by the respective models. The resulting estimates should thus be regarded as model-derived statistical associations rather than demonstrated causal effects. In particular, the changes in estimated risk under hypothetical interventions on ABI, IL-6, or SNCV do not represent the benefits that would be expected from treating these variables in clinical practice. Further prospective and controlled studies are needed to determine whether changes in these biological measures are associated with subsequent changes in DPN trajectories.

## 5 Conclusions

This prospective cohort study identified three distinct 24-month clinical trajectories among patients with DPN receiving TENS. ABI, IL-6, and SNCV showed consistent associations with persistent high symptom burden across the analyses.

The findings indicate that vascular, inflammatory, and neurophysiological measures were associated with differences in long-term clinical trajectories. These associations do not establish causal relationships or treatment effects. Validation in independent cohorts and prospective controlled studies is needed to determine whether these measures have practical value for identifying patients at risk of persistent symptom burden.

## Data Availability

The raw data supporting the conclusions of this article will be made available by the authors, without undue reservation.

## Supplementary material

Supplementary Figure 1 Sensitivity analysis of trajectory stability and mathematical coupling. Sensitivity analysis using an electrophysiology-free outcome (ΔGCS_exSNCV_) to assess the stability of LCGA-derived trajectories and the potential influence of mathematical coupling between SNCV and GCS. **(A)** Trajectory Comparison Across Outcome Definitions. Spaghetti plots and fitted mean trajectories over 24 months for the primary GCS model and the electrophysiology-free model. Both models identified three comparable trajectory patterns: Class 1 (Favorable Improvement, 29.9%), Class 2 (Moderate Improvement, 42.0%), and Class 3 (Persistent High Burden, 28.0%). **(B)** Classification Agreement. Cross-tabulation of trajectory assignments between the two models. Overall agreement was 92.6% (597/645), with a Cohen’s *κ* of 0.915 (95% CI, 0.885–0.945). **(C)** Baseline SNCV and Trajectory Membership. Forest plot of univariable odds ratios (ORs) with 95% CIs for baseline clinical variables associated with trajectory membership. Lower baseline SNCV was associated with Class 3 membership (OR, 0.87; 95% CI, 0.82 – 0.92; *P* < 0.001). **(D)** Multivariable Analysis of Baseline SNCV. Unadjusted and adjusted associations of baseline SNCV with Class 3 membership in the primary and electrophysiology-free models. The adjusted estimates were similar between models (OR, 0.88 vs. 0.87), indicating that the association of baseline SNCV with persistent symptom burden was largely preserved after removing SNCV from the composite outcome. **(E)** Summary of Sensitivity Findings. Comparison of trajectory classification and baseline SNCV associations across the two outcome specifications, supporting the stability of the trajectory structure and reducing concern that the primary findings were driven solely by mathematical coupling. CI, confidence interval; GCS, Global Composite Score; LCGA, latent class growth analysis; OR, odds ratio; SNCV, sensory nerve conduction velocity.
