# Supplementary material for: Longitudinal clinical trajectories and vascular–inflammatory–neurophysiological associations in diabetic peripheral neuropathy: an exploratory analysis of a TENS-treated cohort
Source: Front Endocrinol (Lausanne). 2026 Sep 3;17:1931594. doi: 10.3389/fendo.2026.1931594 (PMC13581509; doi:10.3389/fendo.2026.1931594)
Supplement: Supplementary file 1 [file SupplementaryFile1.docx]

****Supplementary Material****

**Figure S1.** Sensitivity Analysis of Trajectory Stability and Mathematical Coupling

Sensitivity analysis using an electrophysiology-free outcome (ΔGCS_exSNCV_) to assess the stability of LCGA-derived trajectories and the potential influence of mathematical coupling between SNCV and GCS.

**(A)** Trajectory Comparison Across Outcome Definitions. Spaghetti plots and fitted mean trajectories over 24 months for the primary GCS model and the electrophysiology-free model. Both models identified three comparable trajectory patterns: Class 1 (Favorable Improvement, 29.9%), Class 2 (Moderate Improvement, 42.0%), and Class 3 (Persistent High Burden, 28.0%).

**(B)** Classification Agreement. Cross-tabulation of trajectory assignments between the two models. Overall agreement was 92.6% (597/645), with a Cohen’s *κ* of 0.915 (95% CI, 0.885–0.945).

**(C)** Baseline SNCV and Trajectory Membership. Forest plot of univariable odds ratios (ORs) with 95% CIs for baseline clinical variables associated with trajectory membership. Lower baseline SNCV was associated with Class 3 membership (OR, 0.87; 95% CI, 0.82 – 0.92; *P* < 0.001).

**(D)** Multivariable Analysis of Baseline SNCV. Unadjusted and adjusted associations of baseline SNCV with Class 3 membership in the primary and electrophysiology-free models. The adjusted estimates were similar between models (OR, 0.88 vs. 0.87), indicating that the association of baseline SNCV with persistent symptom burden was largely preserved after removing SNCV from the composite outcome.

**(E)** Summary of Sensitivity Findings. Comparison of trajectory classification and baseline SNCV associations across the two outcome specifications, supporting the stability of the trajectory structure and reducing concern that the primary findings were driven solely by mathematical coupling.

**Abbreviations**: **CI**, confidence interval; **GCS**, Global Composite Score; **LCGA**, latent class growth analysis; **OR**, odds ratio; **SNCV**, sensory nerve conduction velocity.
